# Pyruvic Oxime Nitrification and Copper and Nickel Resistance by a *Cupriavidus pauculus*, an Active Heterotrophic Nitrifier-Denitrifier

**DOI:** 10.1155/2014/901702

**Published:** 2014-12-15

**Authors:** Miguel Ramirez, Jennifer Obrzydowski, Mary Ayers, Sonia Virparia, Meijing Wang, Kurtis Stefan, Richard Linchangco, Domenic Castignetti

**Affiliations:** Department of Biology, Loyola University of Chicago, 1032 West Sheridan Road, Chicago, IL 60626, USA

## Abstract

Heterotrophic nitrifiers synthesize nitrogenous gasses when nitrifying ammonium ion. A *Cupriavidus pauculus*, previously thought an *Alcaligenes* sp. and noted as an active heterotrophic nitrifier-denitrifier, was examined for its ability to produce nitrogen gas (N_2_) and nitrous oxide (N_2_O) while heterotrophically nitrifying the organic substrate pyruvic oxime [CH_3_–C(NOH)–COOH]. Neither N_2_ nor N_2_O were produced. Nucleotide and phylogenetic analyses indicated that the organism is a member of a genus (*Cupriavidus*) known for its resistance to metals and its metabolism of xenobiotics. The microbe (a *Cupriavidus pauculus* designated as *C. pauculus* strain UM1) was examined for its ability to perform heterotrophic nitrification in the presence of Cu^2+^ and Ni^2+^ and to metabolize the xenobiotic phenol. The bacterium heterotrophically nitrified well when either 1 mM Cu^2+^ or 0.5 mM Ni^2+^ was present in either enriched or minimal medium. The organism also used phenol as a sole carbon source in either the presence or absence of 1 mM Cu^2+^ or 0.5 mM Ni^2+^. The ability of this isolate to perform a number of different metabolisms, its noteworthy resistance to copper and nickel, and its potential use as a bioremediation agent are discussed.

## 1. Introduction

Heterotrophic nitrification is the oxidation of nitrogenous compounds, both organic and inorganic, to more oxidized products. Organic nitrogenous compounds, for example, may be oxidized to organic nitrocontaining compounds or can be converted to NO_2_^−^ or NO_3_^−^ [1, 2]. Denitrification, the reduction of the nitrogen oxides NO_3_^−^ and NO_2_^−^ to N_2_O or N_2_ [3], is performed by a diverse group of prokaryotes, including members of the* Archaea* and the* Bacteria* [4, 5]. Castignetti and Hollocher [6] described a bacterium, provisionally identified as an* Alcaligenes* sp., which was capable of the two processes, that is, both vigorous heterotrophic nitrification and denitrification. They also noted that a number of different denitrifiers, representing some of the more commonly encountered soil denitrifying microbes [4, 7], heterotrophically nitrify [1].

Heterotrophic nitrification is performed by chemoheterotrophs [2, 7]. The process has the potential to result in nitrogen oxidation [2, 8] and to effect bioremediation, especially with respect to the removal of excess nitrogen [2, 9]. Jetten et al. [2] noted that* Thiosphera pantotropha* (now* Paracoccus pantotrophus*) and other heterotrophic nitrifiers could simultaneously oxidize and reduce N compounds with gaseous N products being formed. Similarly, heterotrophic nitrifiers have been noted to aerobically denitrify [10]. The simultaneous processes of heterotrophic nitrification and denitrification were postulated to have resulted in the underestimation of the significance of heterotrophic nitrification [2, 11]. Rather than inorganic N sources, organic N sources were the substrates for heterotrophic nitrification, which, in some environments, was more significant than autotrophic nitrification [11, 12].

The role of heterotrophic nitrifiers is the subject of current research [13–17] as such microbes may be of consequence to both natural (pastures and grasslands) [8, 9, 11, 12] and artificial (wastewater treatment) [2, 10, 13, 18] environments. Some of these organisms have significant capacities to both heterotrophically nitrify and denitrify [2, 10, 19] and some synthesize N_2_O or N_2_ during the course of heterotrophic nitrification [17, 20] or heterotrophic nitrification-aerobic denitrification metabolism [10, 14–16, 18, 21]. To date, studies have focused on inorganic N compounds as the sources of nitrogenous gas production, with either NH_4_^+^, NH_2_OH, or NO_2_^−^ serving as the substrate for either N_2_ or N_2_O production [10, 14, 16–19]. Whether organic-N substrates, such as pyruvic oxime (PO; H_3_C–C(NOH)–COOH), serve as sources of N_2_ or N_2_O synthesis during heterotrophic nitrification or heterotrophic nitrification coupled to aerobic denitrification has received little, if any, attention.

The current study was undertaken to examine whether the heterotrophic nitrification of PO by the organism previously known as an* Alcaligenes* sp. (1) resulted in the gaseous nitrogen products N_2_ or N_2_O, (2) supported heterotrophic nitrification-aerobic denitrification such that either N_2_ or N_2_O was produced, and (3) occurred when the organism was cultured in the presence of the heavy metals Cu and Ni. In addition, (1) whether a representative xenobiotic, phenol, was used as a carbon source, (2) an analysis of the bacterium's phylogenetic identity, and (3) the identity of the crucial gene necessary for denitrification (nitrite reductase) were conducted. At the time of its original characterization, the* Alcaligenes* sp.'s taxonomic classification was based on phenotypic traits and its G+C content [66.1 mol%-D. Castignetti, D. (1980) Characterization of a soil heterotrophic nitrifier and its synergistic interactions with* Nitrobacter agilis*. Dissertation, University of Massachusetts, Amherst, MA]. In the current study, the bacterium was characterized with respect to its 16S rRNA gene sequence and nitrite reductase (*nir*) gene via cloning and nucleotide sequencing. Identification of the bacterium as a* Cupriavidus pauculus* spurred an examination of its Cu^2+^ and Ni^2+^ resistance capacities, its ability to heterotrophically nitrify PO in the presence of these metals, and its use of the xenobiotic phenol in the absence or presence of Cu^2+^ and Ni^2+^ as this genus is known for its tolerance of metals and use of xenobiotics. (To avoid confusion, the provisionally identified* Alcaligenes* sp. will be referred to as* C. pauculus* UM1 in the remainder of this report.)

## 2. Materials and Methods

### 2.1. Resting Cell Experiments

To examine the ability of* C. pauculus* UM1 to produce nitrogenous gasses while heterotrophically nitrifying PO, the bacterium was grown to mid-to-late logarithmic phase in the PO-mineral salts medium described below, harvested by centrifugation (10,000 ×g for 10 min) and washed twice in 50 mM pH 7.0 phosphate buffer to make resting cells. Cells were divided into control (boiled for 5 minutes) and experimental groups and 5 mL was placed into a 33 mL vial along with a stirring bar. Vials were then sealed with a rubber septum and flushed, for 5 min, with the appropriate gas (see below) to remove the air. At *t* = 0, 1 mL of 60 mM PO in the phosphate buffer was added via syringe and needle to yield a final concentration of 10 mM PO. Gasses used to flush the vials were either 100% helium (He) or 100% O_2_. Three sets of conditions were examined: no O_2_ (anaerobic), low O_2_ (3.6% O_2_), and 100% O_2_. Helium was used to flush the vials either when an anaerobic environment was to be tested or when 1 mL of pure O_2_ was added, via syringe and needle, to yield an approximately 3.6% O_2_ atmosphere in the vials. During PO metabolism by the organism, the oxidation of PO-nitrogen results in its conversion to NO_2_^−^ [22]. NO_2_^−^ synthesis from PO was therefore used to monitor PO use throughout these experiments. At *t* = 0, approximately 3 and 22 hours, 0.11 mL of cells was removed from each vial (via a syringe and needle) and used to measure the consequent production of NO_2_^−^ from PO. 40 *μ*L of gaseous headspace was simultaneously removed to measure the synthesis of N_2_, NO, N_2_O, and CO_2_ by the cells. As* C. pauculus* UM1 is a respiratory microbe, it was not expected that cells without O_2_ (He only) would nitrify. Hence, to vials that received only He, 10 mM NaNO_2_ was added to ensure the presence of an oxidant. This condition allowed for assessment of heterotrophic nitrification of the PO at the expense of NO_2_^−^ reduction, in which case the NO_2_^−^ would be the oxidant and substitute for O_2_ gas.

### 2.2. Growth and Metal Tolerance

To determine the bacterium's tolerance of either Cu^2+^ or Ni^2+^, growth was performed using mid-to-late log cultures as the inoculum (0.5% (v/v)) of the bacterium grown in nutrient broth (NB-Sigma Chemical Co., St. Louis, MO) without any metal added. To perform a growth curve with Cu and Ni present, the medium used was NB to which the metals were added from 2 M stocks of either CuCl_2_–2H_2_O or NiCl_2_–6H_2_O to result in the desired final concentrations [23]. Controls consisted of NB devoid of metal additions. Minimum inhibitory concentrations (MIC) experiments were conducted as described by Chess [24] using NB supplemented with Cu^2+^ or Ni^2+^ rather than antibiotics. For both growth and MIC experiments, samples were run in triplicate and each experiment was performed at least twice. For all growth experiments, cultures were incubated at 28–30°C and shaken at 300 r.p.m.

To determine* C. pauculus* UM1's ability to heterotrophically nitrify PO when either Cu^2+^ or Ni^2+^ was present, three different types of growth experiments were conducted. For the first, the bacterium was cultured in NB with 10–12 mM PO with either 1 mM Cu^2+^ or 0.5 mM Ni^2+^ and PO use was monitored as described above (via conversion to NO_2_^−^). Controls consisted of identical cultures but without PO. To determine if the organism could heterotrophically nitrify PO without supplemental carbon nutrients (i.e., NB), a second experiment type was used where the* C. pauculus* UM1 was grown in a mineral salts medium, previously described [25], containing either 1 mM Cu^2+^ or 0.5 mM Ni^2+^, in which 10–12 mM PO served as the sole C source and where the uninoculated medium was the control. The minerals salts medium was made and autoclaved without 0.5 g L^−1^ MgSO_4_-7H_2_O, 0.5 g L^−1^ NH_4_Cl, and the 10–12 mM PO, which were filter-sterilized and added to the cooled, sterile medium. To determine the effect of Cu^2+^ and Ni^2+^ on the relative rate of* C. pauculus* UM1's ability to grow and nitrify PO, the third experiment type was conducted in which* C. pauculus* UM1 was grown in the above mineral salts medium without metals (control) or with either 1 mM Cu^2+^ or 0.5 mM Ni^2+^. To determine if* C. pauculus* UM1 could use phenol as a sole source of carbon, the organism was grown to midlog in NB and used at a 1 : 100 dilution to inoculate the above mineral salts medium containing filter-sterilized 0.5 g L^−1^ MgSO_4_-7H_2_O, 0.5 g L^−1^ NH_4_Cl, and phenol (2 mM) as the sole C source. Culturing of* C. pauculus* UM1 into this medium lacking phenol showed that “carryover” growth of the bacterium due to residual NB did not occur as growth measurements noted a decline of culture turbidity with time as opposed to an increase if phenol was present.

To determine if* C. pauculus* UM1 could use phenol as a sole C source when cultured in the presence of either 1 mM Cu^2+^ or 0.5 mM Ni^2+^, the bacterium was grown in ~2 mM phenol-mineral salts medium and used at a 1 : 100 dilution to inoculate the same medium containing either no metals, 1 mM Cu^2+^, or 0.5 mM Ni^2+^. As the medium with 1 mM Cu^2+^ had an initial absorbance above 0 and growth with phenol was fairly sparse, growth was spurred in the cultures by adding an aliquot of filter-sterilized 200 mM phenol solution after 7 days, thereby reestablishing the cultures to a phenol concentration of ~2 mM.

### 2.3. Nucleotide Cloning, Sequencing, and Gene Identifications

The subject of investigation is a bacterium originally isolated from an agricultural soil [22, 25]. The 16S rRNA gene sequence of the bacterium was analyzed by conducting PCR using the* Escherichia coli* equivalents from base pair 8 to base pair 926 and then from base pair 906 to 1512. A total of 1501 nucleotides of the bacterium's 16S rRNA gene were cloned and sequenced.

Genomic DNA from the bacterium, for both 16S rRNA gene and* nirS* and* nirK* gene analyses (see below), was isolated using the Wizard Genomic DNA Purification Kit (Promega, Madison, WI) and used as template DNA. The first primer pair employed was 8F and 926R, the 8F primer sequence being 5′ AGAGTTTGATCCTGGCTCAG 3′ [26]. The complement to the 8F primer is the 926R primer which has the sequence of 5′ CCGTCAATTCCTTTRAGTTT 3′ [27]. The second primer pair consists of 906F and 1512R. The 1512R sequence was 5′ ACGGYTACCTTGTTACGACTT 3′ [28] while the 906F sequence is the 5′ to 3′ reverse complement of the 926R primer (16S rDNA). PCR reactions were performed using either a Hybaid PCR Sprint Thermocycler (Cole-Parmer, Vernon Hills, IL) or a Techne-412 (Scie-Plas Ltd., Cambridge, UK) thermocycler. PCR Master Mix (Promega, Madison, WI) was used, as described by the manufacturer to make 50 *μ*L samples. PCR conditions were one cycle of 94°C for 4 min followed by 33 cycles of 94°C for 0.75 min, 56.4°C for 1 min, and 72°C for 1.5 min followed by one cycle of 72°C for 7 min. Samples were then kept at either 4 or 10°C until removed for analysis. Gel analysis was routinely performed using a sodium borate system [29]. Negative controls, with sterile water substituted for template DNA (water controls), were performed to ensure that the DNA copied was from the bacterium being analyzed. Genomic DNA of stock bacteria (e.g.,* Pseudomonas* spp.,* E. coli*) was used as a positive control to insure that amplicons of the proper size were made.

Amplicons of interest were cloned as described in the instructions for the TOPO TA Cloning Kit for Sequencing system (Invitrogen, Carlsbad, CA). The resulting pCR4 plasmids, containing the amplicons of interest, were isolated using the Wizard Plus SV Minipreps DNA Purification System (Promega, Madison, WI), verified that they contained the clone of interest by using the plasmid to produce the appropriate PCR product, and then sequenced by the University of Chicago Cancer Research Center DNA Sequencing Facility (Chicago, IL) using either an Applied Biosystems 3730XL 96-capillary or a 3130 16-capillary automated DNA sequencer. Sequences were analyzed using Chromas Lite (http://www.technelysium.com.au/chromas_lite.html Technelysium Pty, Ltd.), to make an initial decision as to the quality and acceptability of the sequence, followed by a more thorough analysis to align and append sequences using Lasergene (DNASTAR, Inc. Madison, WI) software. Analysis of the 16S rRNA gene nucleotide sequence allowed for the construction of appended nucleotide sequences of the* E. coli* equivalent of base pairs 8-1512. Sequences were aligned, appended, and analyzed via BLAST-N (GenBank-National Center for Biotechnology Information, http://www.ncbi.nlm.nih.gov/) to search for matches and to identify the closest sequenced relatives. The data generating the best matches were used to characterize the bacterium. In addition, the validity and the quality of the GenBank 16S rRNA gene sequences were investigated via submitting the sequences to Greengenes [30]. Sequence alignments and phylogenetic analysis of the bacterium were performed via the use of the SeaView (http://pbil.univ-lyon1.fr/software/seaview.html) software [31]. Phylogenetic tree construction was done via the SeaView program using the parameters of 100 bootstrap replicates and the NJ algorithm. The dissimilarity bar and accession numbers of the sequences used for comparison are included in the figure.

Denitrifying bacteria contain the crucial key enzyme (nitrite reductase), which is necessary to reduce nitrite to nitric oxide [3, 32, 33]. A denitrifying bacterium contains nitrite reductase as either the cytochrome cd_1_ (NirS) or the copper-containing (NirK) enzyme and an individual organism contains a single type of the nitrite reductase gene, either* nir*S or* nirK* [3, 32, 33]. As* C. pauculus* UM1 had previously been identified as an active denitrifier [6], we examined it for the presence of either the* nir*S or* nirK* gene by using the primer pairs and amplification conditions described by either Braker et al. [32] or Throbäck et al. [33]. The only modification was that the annealing temperature of the Throbäck et al. [33] procedure was adjusted to 57°C. For the examination of the presence of the* nirS* gene, the primer sets were* nirS*1F and* nirS*6R [32] or cd3aF and Rc3d [33]. The former yields a theoretical 873 bp amplified product while the latter yields a theoretical amplified product of 425 bp. Primer sets for the examination of the presence of the* nirK* gene were* nirK*1F and* nirK*5R [32] or FlaCu and R3Cu [33]. The former yields a theoretical 514 bp amplified product while the latter yields a theoretical amplified product of 475 bp. DNA from* Pseudomonas stutzeri* (ATCC 11607) served as a positive control for the amplification of the* nirS* amplicon while DNA from* Achromobacter xylosoxidans* (ATCC 15173) served as a positive control for the amplification of the* nirK* amplicon. Once the amplicon of the correct size for* nirS* was identified (the bacterium contains only the* nirS* gene—see below), it was cloned using the TOPO TA Cloning Kit for Sequencing system noted above. The resultant plasmid was isolated, verified via PCR as containing a* nirS* amplicon, and sequenced. Aligned and appended sequences were analyzed by BLAST-N to identify the closest matches.

### 2.4. Nucleotide Sequences

The partial 16S rRNA sequence of the bacterium and the* nirS* gene sequences have been deposited in GenBank and are listed below.

### 2.5. Chemicals and Analytic Methods

All chemicals used to make the above media were of reagent grade or better. The sodium salt of PO was synthesized via the method of Quastel et al. [34]. NO_2_^−^ synthesis was measured to monitor PO usage and was determined by the sulfanilamide-N-(1-naphthyl)ethylenediamine method [35]. Molecular sieve (Linde-Union Carbide, South Plainfield, NJ) and Poropak Q (Waters, Milford, MA) columns, in a Shimadzu GC-8A (Kyoto, Japan) gas chromatograph, were used to determine CO_2_, N_2_, NO, and N_2_O as described [36]. Growth was measured via absorbance at 600 nm and phenol was quantified via the method of Slinkard and Singleton [37], adjusted to accommodate 1 mL samples.

### 2.6. Statistical Analyses

Comparison of mean values (Tables 1 and 2 and Figure 3) was performed via *t*-testing using SYSTAT 13 or Excel. Comparison of the curves and of the individual points (means of three replicates) for a given time in Figures 2 and 3 was done using the ANOVA and Tukey's honestly significantly different tests in SYSTAT 13.

## 3. Results and Discussion

To determine if the bacterium produces either N_2_ or N_2_O while heterotrophically nitrifying, we attempted to grow the bacterium in sealed test tubes from which gasses could be sampled. Due to insufficient growth of the bacterium, these experiments were discontinued.* C. pauculus* UM1 was thus grown in the mineral salts-PO medium aerobically at 30°C, harvested at mid-to- late logarithmic growth and resting cells were prepared. Under either 0% (He), 3.6%, or 100% O_2_ atmospheres, resting cells of the bacterium produced neither N_2_, NO nor N_2_O. Although control (boiled) vials failed to produce either NO_2_^−^ or CO_2_, experimental vials clearly produced NO_2_^−^ by the 3 hours and CO_2_ was noted by the 22-hour time point.

A number of organisms produce either N_2_ or N_2_O while heterotrophically nitrifying or during heterotrophic nitrification-aerobic denitrification [2, 14, 16, 17, 20]. When PO was present under anaerobic conditions, with NO_2_^−^ present to serve as an oxidant, neither N_2_ nor N_2_O was synthesized. With PO as a nitrogen source and under full or limited O_2_ conditions,* C. pauculus* UM1 produced neither N_2_ nor N_2_O but did make NO_2_^−^, indicating that while heterotrophic nitrification had occurred, aerobic, or anaerobic, denitrification had not. While previously shown to be clearly capable of heterotrophic nitrification ([22, 25] and this study) and denitrification [6], the current experiments indicate that* C. pauculus* UM1 produced neither N_2_ nor N_2_O via heterotrophic nitrification or via heterotrophic nitrification-aerobic denitrification.

NH_3_ or NH_4_^+^ are the likely sources of N_2_ and N_2_O produced via heterotrophic nitrification [2, 16, 17, 20]. The failure of* C. pauculus* UM1 to make either of these gasses likely reflects that PO oxidation to NO_2_^−^ probably occurs via an organic route, that is, via a carbon intermediate and not via NH_2_OH [34, 38, 39]. While* C. pauculus* UM1 vigorously denitrifies [6] and avidly oxidizes both the organic-N source PO and the inorganic N-source hydroxylamine [22, 25], the inability of* C. pauculus* UM1 to synthesize either N_2_ or N_2_O during the oxidation of PO limits its ability to remove N as a gas from aerobic environments.

**Figure 1:**
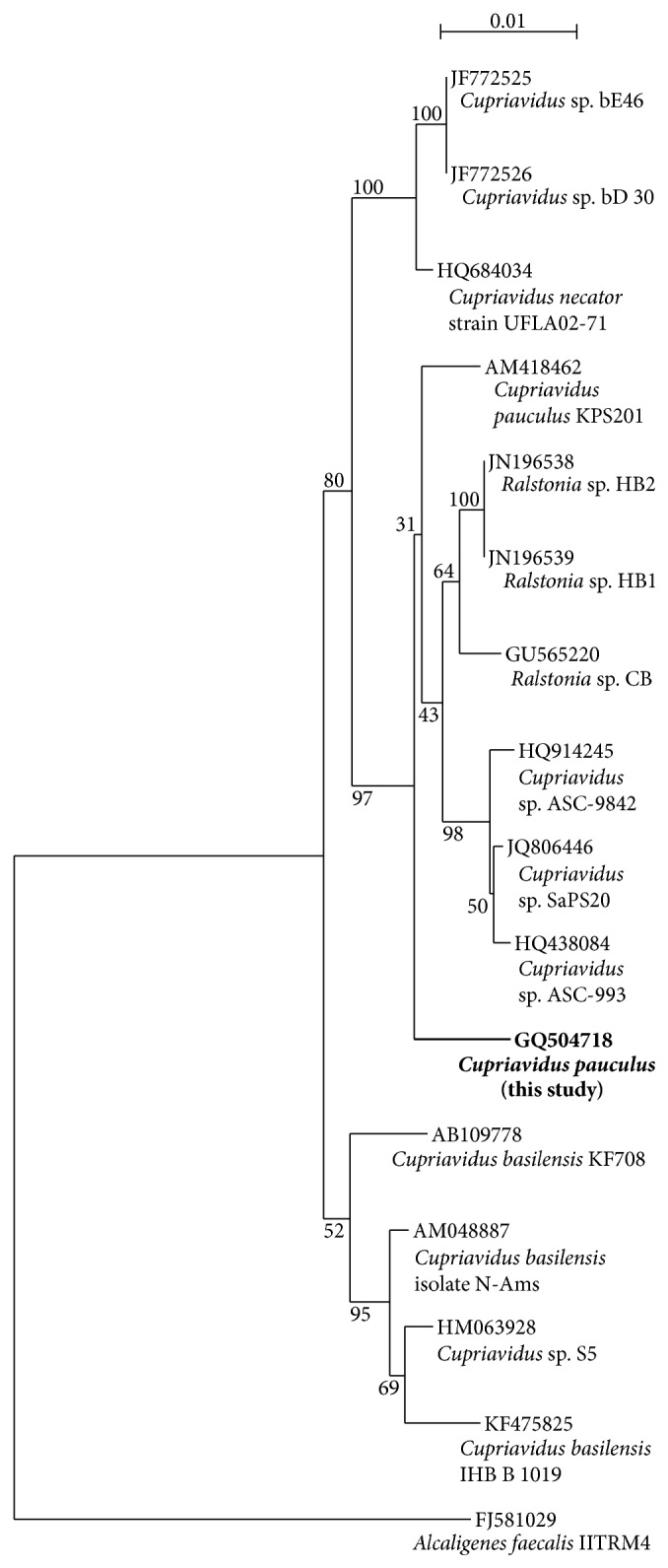
Phylogenetic analysis of the bacterium of this study based on 16S rRNA gene homologies. (The species/strain designations used for comparison are included in the figure.* Alcaligenes faecalis* IITRM4 served as the outgroup.)

As the bacterium was originally described prior to today's nucleotide sequencing techniques, the phylogenetic identities of the bacterium and the nitrite reductase gene of the organism were of interest. PCR primers [40] specifically designed to amplify 16S rRNA genes from* Ralstonia* (i.e.,* Ralstonia* and* Cupriavidus*, see below) resulted in an amplicon of the proper size from genomic DNA of the bacterium but not from genomic DNA from a non-*Cupriavidus* (*Pseudomonas*) bacterium. The cloning, sequencing, and BLAST-N analysis of 1501 nucleotides of* C. pauculus* UMI's 16S rRNA gene resulted in the greatest similarity (98.0% identity) match to* Cupriavidus pauculus* strain KPS201 and* Ralstonia* sp. HB1 and HB2. A similar match (97.9%) to* C. pauculus* strain KPS201 was also obtained from Greengenes. A multiple alignment of similar 16S ribosomal DNA sequences indicates that the bacterium is most closely related to* C*.* pauculus* KPS201 and other* Cupriavidus* species (Figure 1). The nucleotide sequence of the 1501 nucleotides of* C. pauculus* UM1's 16S rRNA gene has been deposited in GenBank under the accession number GQ504718. Of the first 24 GenBank matches, including 20 of which were noted as being members of the genera* Cupriavidus* and* Ralstonia* or the family Burkholderiaceae (of which* Cupriavidus* (*Ralstonia*) are included genera—the other matches were to unidentified isolates), the sequence is a perfect match between nucleotides 906–926, strongly indicating that the primers used for this region did not result in errant sequence data.

A 98% identity match to a species' 16S rRNA nucleotide sequence is considered species identification [41, 42]. Analyses of the 16S rRNA gene sequence of* C. pauculus* UM1 revealed that its greatest homology was to the microbes* C. pauculus* strain KPS201 and* Ralstonia* sp. HB1 or HB2, all of which had a GenBank 98.0% similarity match and *E* values of 0.0. Nucleotide analysis by Greengenes (the more reliable database as it screens for chimeras and performs standard alignments and taxonomic classifications based on multiple published taxonomies [30]) indicated a 97.9% match to* Cupriavidus pauculus* strain KPS201, with the second closest match being to* Ralstonia eutropha* strain H16 (97.4%).

We note that* Alcaligenes eutrophus* has been reclassified as* Ralstonia eutrophus*, then as* Wautersia eutropha*, and finally as* Cupriavidus necator*, the type species [43].* Cupriavidus pauculus* is now the accepted nomenclature for the microbes previously known as* Ralstonia paucula* and* Wautersia paucula* [43]. As the* Ralstonia* spp. bear no species identification and are thus of less consequence than the bacterium (*C. pauculus* KPS201) identified as being the closest match in Greengenes, we propose that the identity of our isolate is a* C. pauculus* strain (*C. pauculus* UM1). Phylogenetic analysis (Figure 1) suggests a close association with* C. pauculus* KPS201 and with organisms originally classified as either* Ralstonia* or* Cupriavidus*. The claim is supported by the bacterium's phenotypic traits [25] and its G+C content (66.1 mol%) as the genus has a G+C content ranging from 63 to 69 mol% [43].

Both of the primer sets used to generate nitrite reductase amplicons yielded highly similar matches to sequences of the* nirS* gene, that is, the cytochrome cd_1_ (*nirS*) nitrite reductase gene (data not shown).* nirS* gene sequences GQ504717, generated from the Braker et al. [32] amplicon, and GQ504716, from the Throbäck et al. [33] amplicon, have been deposited in GenBank.

The ability to denitrify is widespread and precludes identifying denitrifiers by using 16S rRNA gene analyses [3, 44, 45]. That the genus* Cupriavidus* is capable of denitrification is supported by work [46] that noted that members of the genus were among the most frequently isolated denitrifiers obtained from a luvisol, that the type species of the genus (*C. necator*) was specifically identified as a denitrifier, and that a number of previously recognized genera capable of denitrification are now part of the* Cupriavidus* genus. GenBank lists 20* Cupriavidus nirS* entries, 9* norB* entries, and 6* nosZ* entries and Schwartz et al. [47] noted that* Ralstonia eutropha* (*C. necator*) H16 contained the megaplasmid pHG1, which had a gene cluster for denitrification. The cloned sequences of* C. pauculus* UM1's nitrite reductase gene were good matches (87–95%) to known* nirS* nucleotide sequences (87%* Ralstonia eutropha* JMP134 chromosome 2, complete sequence, 95%* Pseudomonas chloritidismutans* partial* nirS* gene for nitrite reductase, isolate AW-1). As denitrifiers contain a single type of nitrite reductase [3, 32, 33] and since our isolate contained a* nirS*-like gene, we conclude that* C. pauculus* UM1 denitrifies via its cytochrome cd_1_ nitrite reductase. While an earlier study [48] reported that* R. paucula* (now* C. pauculus*) was unable to denitrify, considering our isolate's resistance to Cu and Ni (see below), its sharing of a denitrification capacity with other members of the genus [46, 47, 49], its 16S rRNA gene homology to* C. pauculus* KPS201, and other* Cupriavidus* spp. and the above G+C content and phenotypic similarities, the data support that* C. pauculus* UM1 denitrifies via the use of a* nirS*-like gene. Of particular interest, and revealed in this study, is that even though* C. pauculus* UM1 sp. contains a* nirS*-like gene which it uses to denitrify [6], the organism did not invoke its use when heterotrophically nitrifying as evidenced by the lack of either N_2_O or N_2_ synthesis under the heterotrophic nitrification or heterotrophic nitrification-aerobic denitrification conditions of the current study.

**Figure 2:**
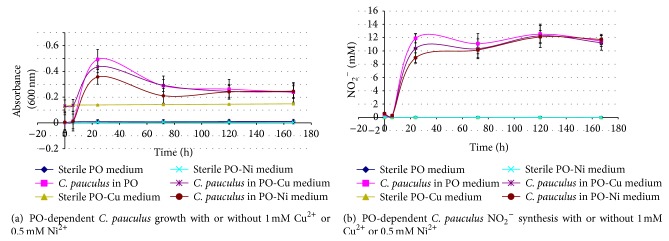
Growth (a) and nitrification (b) of* C. pauculus* UM1 in PO-mineral salts medium with or without either 1.0 mM Cu^2+^ or 0.5 mM Ni^2+^. (The inoculum for the cultures was grown in 0.2% PO-mineral salts medium until mid-to-late logarithmic growth and used at 0.5% (v/v). The growth curves for* C. pauculus* UM1 either with 1.0 mM Cu^2+^ (*P* ≤ 0.05) or 0.5 mM Ni^2+^ (*P* ≤ 0.01) or without Cu^2+^ or Ni^2+^ (*P* ≤ 0.01) were significantly different from their corresponding uninoculated controls. Similarly, the NO_2_^−^ synthesis curves either with 1.0 mM Cu^2+^, 0.5 mM Ni^2+^ or without either Cu^2+^ or Ni^2+^ were significantly different (*P* ≤ 0.01) from their corresponding uninoculated controls. At the *T* = 24 and 72 hours, growth of all inoculated* C. pauculus* UM1 cultures was significantly different (*P* ≤ 0.01) than the corresponding uninoculated controls. In addition, at *T* = 120 and 167 hours,* C. pauculus* UM1 cultures grown either without Cu^2+^ or Ni^2+^ or with 0.5 mM Ni^2+^ were significantly different (*P* ≤ 0.01) than their corresponding uninoculated controls. At *T* = 24 hours, the growth of* C. pauculus* UM1 with 0.5 mM Ni^2+^ was significantly less (*P* ≤ 0.01) than* C. pauculus* UM1 grown without Cu^2+^ or Ni^2+^. At *T* = 120 and 167 hours, the growth of all* C. pauculus* UM1 cultures was not significantly different than the uninoculated 1 mM Cu^2+^ control. At all measurements, NO_2_^−^ concentrations were significantly different (*P* ≤ 0.01) between the inoculated cultures and their corresponding uninoculated controls. It is presumed that, at the early time points (*T* = 0 and perhaps 6 hours), this was due to NO_2_^−^ carryover with the inocula. At *T* = 24 hours, NO_2_^−^ concentrations were significantly different (*P* ≤ 0.05) between the inoculated PO-0.5 mM Ni^+^ cultures and inoculated PO cultures without either Cu^2+^ or Ni^2+^.) Error bars represent the standard deviation of a point.

Makkar and Casida [50] were the first to characterize the genus* Cupriavidus.* They noted that the type strain (*C. necator*) grew more quickly in media containing 0.4, 0.6, or 0.8 mM Cu^2+^ than with no copper. The genus is noted for its metal resistance [43] and members have been studied for their ability to tolerate metals [51, 52]. Like* C. necator*,* C. pauculus* UM1 tolerated copper (and nickel) well, demonstrating robust growth in media containing 1 mM Cu^2+^ and 0.5 mM Ni^2+^ (Tables 1 and 2 and Figure 2). MIC values were determined as 3 mM Cu^2+^ and 1 mM Ni^2+^ (data not shown).* C. pauculus* UM1 also grew readily and heterotrophically nitrified well, when in NB-PO with either 1 mM Cu^2+^ or 0.5 mM Ni^2+^ (Table 1). Confirmation of* C. pauculus* UM1's ability to withstand Cu^2+^ was noted as it grew readily in the 1 and 2 mM Cu^2+^ synthetic medium used to study the related bacterium* Cupriavidus metallidurans* CH34 [51].

The ability of* C. pauculus* UM1 to heterotrophically nitrify PO in the absence of other carbon sources (no NB present) when either Cu^2+^ or Ni^2+^ was present was investigated. When in the presence of either 1 mM Cu^2+^ or 0.5 mM Ni^2+^, the bacterium readily used PO as its sole C source, nitrifying it vigorously (Table 1). The relative ability of the bacterium to use PO and to nitrify it with and without the metals was also examined (Figure 2).* C. pauculus* UM1 grew well and nitrified readily under these conditions, demonstrating growth and nitrification when in the presence of either 1 mM Cu^2+^ or 0.5 mM Ni^2+^ that was commensurate with the metals being absent. The higher Abs_600_ of either the control or experimental samples containing Cu^2+^ was due to the blue color imparted to the clear medium by the 1 mM Cu^2+^.

*C. pauculus* KPS 201 [23] was able to withstand notably high concentrations of nickel (MIC of 28.9 mM). More common metal resistance values of approximately 0.5–12 mM Cu [53–55] and 2–25 mM Ni [53, 54, 56, 57] have been noted for a number of metal-resistant microbes, with Ni-sensitivity cited as tolerating no more than 0.1 mM Ni [57].* C. pauculus* UM1 readily grew in media containing either 1 mM Cu^2+^ or 0.5 mM Ni^2+^ (Tables 1 and 2 and Figure 2) and tolerated higher copper than nickel concentrations (see above).* C. pauculus* UM1 is thus Cu and Ni tolerant when compared to other microbes [53–56]. Of interest is the observation that* C. pauculus* UM1's ability to use PO, as both a carbon and heterotrophic nitrification source, was not impeded by the metals (Table 1 and Figure 2). When an inoculum was grown in a nutrient-rich medium (NB) containing either 1 mM Cu^2+^ or 0.5 mM Ni^2+^ and was used to inoculate media containing either NB-PO, NB-PO-1 mM Cu^2+^, or NB-PO-0.5 mM Ni^2+^,* C. pauculus* UM1 grew as fast in the presence of either 1 mM Cu^2+^ or 0.5 mM Ni^2+^ as without the metals being present (data not shown) and yielded higher amounts of cells (Table 1). When* C. pauculus* UM1 was grown in a PO-mineral salts medium and used to inoculate either PO, PO-1 mM Cu^2+^, or PO-0.5 mM Ni^2+^ mineral salts media, the organism grew as readily in the metal-containing media and nitrified to the same extent, producing equal amounts of NO_2_^−^ (Figure 2).

**Figure 3:**
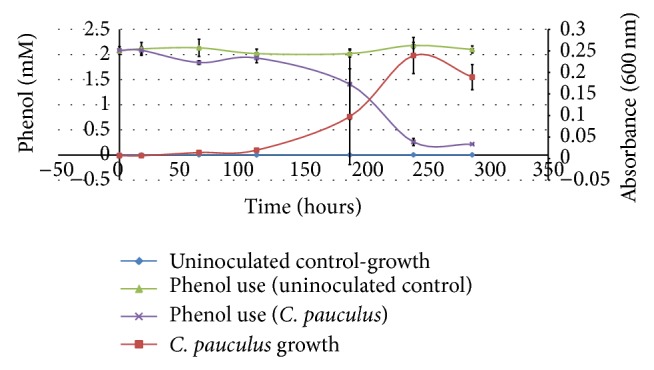
Growth of* C. pauculus* UM1 with phenol as the sole carbon source. (For both the growth of* C. pauculus* UM1 and the amount of phenol remaining in the media, significant differences (*P* ≤ 0.01) were noted for 244 and 288 hours.) Error bars represent the standard deviation of a point.

Xenobiotics metabolized and degraded by* C. pauculus* include benzene, toluene [58], 3-nitrophenol [59], the herbicide 2,4-dichlorophenoxyacetic acid [60, 61], and the pesticides chlorpyrifos (O,O-diethyl O-(3,5,6-trichloro-2-pyridyl)phosphorothioate) and TCP (3,5,6-trichloro-2-pyridinol) [62, 63]. As a representative xenobiotic, phenol (2 mM) supported the growth of* C. pauculus* UM1 (Figure 3), using virtually all of the phenol by the end of the experiment. A shorter induction (about 2-3 days) was observed if the concentration of phenol was reduced to 1 mM (data not shown).* C. pauculus* UM1 also demonstrated the ability to use phenol in the presence of either Cu^2+^ or Ni^2+^ (Table 2) which suggests that it may be an effective bioremediation agent in environments where these metals are present. Preliminary experiments show that* C. pauculus* UM1 grows in media with catechol as the sole C source (data not shown). That* C. pauculus* UM1 uses phenol as a sole carbon source (Table 2 and Figure 3) suggests that catechol is an intermediate as phenol is known to be metabolized via one of two catechol-dependent pathways [64, 65].

This report is the first, to our knowledge, to describe a member of the genus* Cupriavidus* as being a heterotrophic nitrifier.* C. pauculus* UM1 is a potent heterotrophic nitrifier and denitrifier and is able to resist the toxic effects of copper and nickel while heterotrophically nitrifying (Table 1, Figure 2 and MIC values of 3 mM Cu^2+^ and 1.0 mM Ni^2+^). Its use of phenol as a carbon source further expands the remarkable metabolic abilities of the genus and the species. Given* C. pauculus* UM1's metabolic capacities, it will be of interest to determine whether the microbe contains the metal resistance genes noted in other* Cupriavidus* spp. as well as the genes necessary to metabolize xenobiotics such as those noted above. We are investigating the use of such xenobiotics by* C. pauculus* UM1. If* C. pauculus* UM1 does contain such genes, it may be employed as a bioremediation agent. Given the bacterium's resistance to copper and nickel, its metabolism of such xenobiotics may be beneficial in those environments where metal tolerance is either an advantage or a necessity.

## 4. Conclusions

A soil microbe was examined for its ability to produce nitrogenous gasses while heterotrophically nitrifying. The organism nitrified the organic substrate pyruvic oxime to nitrite well but produced neither N_2_ nor N_2_O. Nucleotide and phylogenetic analyses resulted in classifying the bacterium as a member of the genus* Cupriavidus*, thus establishing that this genus can heterotrophically nitrify. The microbe,* C. pauculus* UM1, performed heterotrophic nitrification in the presence of Cu^2+^ and Ni^2+^, nitrifying well when either 1 mM Cu^2+^ or 0.5 mM Ni^2+^ was present.* C. pauculus* UM1 also metabolized the xenobiotic phenol, using it as substrate for growth in either the absence or presence of 1 mM Cu^2+^ or 0.5 mM Ni^2+^.

Subsequent to the submission of this paper, 16S rRNA gene cloning of* C. pauculus* UM1 was performed using the primer pair of Yu et al. (Yu, C, Hongwei, G, Yanming, Z, Mingjun, D, Zhenxying, W, Laihua, Z, Qing, D, Biao, X, Chengzhu, L, Zhiquin, Y, Xizhi X (2012), analysis of the bacterial diversity existing on animal hide and wool: development of a preliminary pcr-restriction fragment length polymorphism fingerprint database for identifying isolates, Journal of AOAC International, 95(6):1750–1754). These primers are designed to clone from base pair 27 to base pair 1492 of the* E. coli* 16S rRNA gene. With respect to base pairs 906–926, the sequence obtained was a perfect match to that previously obtained for* C. pauculus* UM1's 16S rRNA gene, further supporting the conclusion that the match between nucleotides 906 and 926 was not the result of primer sequence resulting in errant sequence data.

## Figures and Tables

**Table 1:** Growth and nitrification of PO by *C. pauculus *UM1 in the presence of either 1 mM Cu^2+^ or 0.5 mM Ni^2+^ in either NB or PO-mineral salts medium^a,b^.

Growth condition	Time (d)			
	0	7	0	7
	Growth (Abs_600_)	Growth (Abs_600_)	NO_2_^−^ (mM)	NO_2_^−^ (mM)
NB-1 mM Cu^2+^	0.01 (0.00)	2.04 (0.09)	0.00 (0.00)	0.01 (0.02)
NB-1 mM Cu^2+^-PO	0.07 (0.00)	2.50 (0.26)	0.00 (0.00)	13.62^d^ (0.48)

NB-0.5 mM Ni^2+^	0.00 (0.00)	1.77 (0.04)	0.00 (0.00)	0.03 (0.01)
NB-0.5 mM Ni^2+^-PO	0.01 (0.00)	2.07^d^ (0.06)	0.00 (0.00)	6.61^d^ (1.29)

PO-1 mM Cu^2+^ (uninoculated)	0.01 (0.01)	0.00 (0.00)	0.00 (0.00)	−0.04 (0.00)
PO-1 mM Cu^2+^	0.00 (0.00)	0.14^d^ (0.01)	0.03^d^ (0.01)	10.39^d^ (0.32)

PO-0.5 mM Ni^2+^ (uninoculated)	0.00 (0.00)	0.00 (0.00)	−0.01 (0.01)	−0.01 (0.00)
PO-0.5 mM Ni^2+^	0.00 (0.00)	0.23^c^ (0.06)	0.12^d^ (0.01)	12.36^d^ (0.98)

^a^Inocula for the NB-1 mM Cu^2+^ and the NB-0.5 mM Ni^2+^ experiments were grown in the NB-1 mM Cu^2+^ and NB-0.5 mM Ni^2+^ medium, respectively, until late logarithmic growth and used at 0.5% (v/v). Inocula for the PO-1.0 mM Cu^2+^ medium and for the PO-0.5 mM Ni^2+^ medium were grown in 0.2% PO medium until late logarithmic growth and used at 0.5% (v/v).

^b^Standard deviation values are in parentheses.

^c^Statistically different from the corresponding control at *P*≤ 0.05.

^d^Statistically different from the corresponding control at *P*≤ 0.01.

**Table 2:** Growth of *C. pauculus *UM1 when in the presence of either no metals, 1 mM Cu^2+^, or 0.5 mM Ni^2+^ in the phenol-mineral salts medium^a,b^.

Growth condition	Time (d)					
	0	3	6	0	3	6
	Growth (Abs_600_)	Growth (Abs_600_)	Growth (Abs_600_)	Phenol (mM)	Phenol (mM)	Phenol (mM)
Phenol	0.0018 (0.0005)	0.0800^d^ (0.0028)	0.0715^d,e^ (0.0038)	1.89 (0.12)	0.27^d^ (0.01)	0.20^d,e^ (0.01)

Phenol with 1 mM Cu^2+^	0.0920 (0.0094)	0.1417^d^ (0.0118)	0.1385 (0.0324)	1.89 (0.12)	0.27^d^ (0.02)	0.22^d,e^ (0.00)

Phenol with 0.5 mM Ni^2+^	0.0025 (0.0005)	0.0769^c^ (0.0252)	0.1793^c^ (0.0616)	1.93 (0.18)	1.30 (0.57)	0.20^d^ (0.00)

^a^Inocula were a 1 : 100 dilution of *C. pauculus* UM1 grown to midlogarithmic phase in 2 mM phenol-mineral salts medium. The relatively large *t* = 0 Abs_600_ value of the phenol with 1 mM Cu^2+^ cultures was due to the blue color imparted to the medium by Cu^2+^.

^b^Standard deviation values are in parentheses.

^c^Statistically different from the initial (time 0) measurement at *P*≤ 0.05.

^d^Statistically different from the initial (time 0) measurement at *P*≤ 0.01.

^e^Statistically different from the day 3 measurement at *P*≤ 0.05.
